# Exosomes derived from platelet-rich plasma administration in site mediate cartilage protection in subtalar osteoarthritis

**DOI:** 10.1186/s12951-022-01245-8

**Published:** 2022-01-29

**Authors:** Yu Zhang, Xiaowei Wang, Jian Chen, Dingfei Qian, Peng Gao, Tao Qin, Tao Jiang, Jiang Yi, Tao Xu, Yifan Huang, Qian Wang, Zheng Zhou, Tianyi Bao, Xuan Zhao, Hao Liu, Ziyang Zheng, Jin Fan, Shujie Zhao, Qingqing Li, Guoyong Yin

**Affiliations:** 1 Department of Orthopedics, The First Affiliated Hospital of Nanjing Medical University, Nanjing, 210029 Jiangsu China; 2 Department of Orthopedics, The Fourth Medical Center, Chinese PLA General Hospital, Haidian, Beijing, 100036 China

**Keywords:** Subtalar osteoarthritis, Platelet-rich plasma, Exosome, In situ drug delivery

## Abstract

Subtalar osteoarthritis (STOA) is often secondary to chronic ankle sprains, which seriously affects the quality of life of patients. Due to its etiology and pathogenesis was not studied equivocally yet, there is currently a lack of effective conservative treatments. Although they have been used for tissue repair, platelet-rich plasma-derived exosomes (PRP-Exo) have the disadvantage of low retention and short-lived therapeutic effects. This study aimed to determine whether incorporation of PRP-Exo in thermosensitive hydrogel (Gel) increased their retention in the joint and thereby playing a therapeutic role on STOA due to chronic mechanical instability established by transecting lateral ligaments (anterior talofibular ligament (ATFL)/calcaneal fibular ligament (CFL)). PRP-Exo incorporated Gel (Exo-Gel) system, composed of Poloxamer-407 and 188 mixture-based thermoresponsive hydrogel matrix in an optimal ratio, was determined by its release ability of Exo and rheology of Gel response to different temperature. The biological activity of Exo-Gel was evaluated in vitro, and the therapeutic effect of Exo-Gel on STOA was evaluated in vivo. Exo released from Exo-Gel continuously for 28 days could promote the proliferation and migration of mouse bone mesenchymal stem cells (mBMSCs) and chondrocytes, at the same time enhance the chondrogenic differentiation of mBMSCs, and inhibit inflammation-induced chondrocyte degeneration. In vivo experiments confirmed that Exo-Gel increased the local retention of Exo, inhibited the apoptosis and hypertrophy of chondrocytes, enhanced their proliferation, and potentially played the role in stem cell recruitment to delay the development of STOA. Thus, Delivery of PRP-Exo incorporated in thermosensitive Gel provides a novel approach of cell-free therapy and has therapeutic effect on STOA.

**Graphical Abstract:**

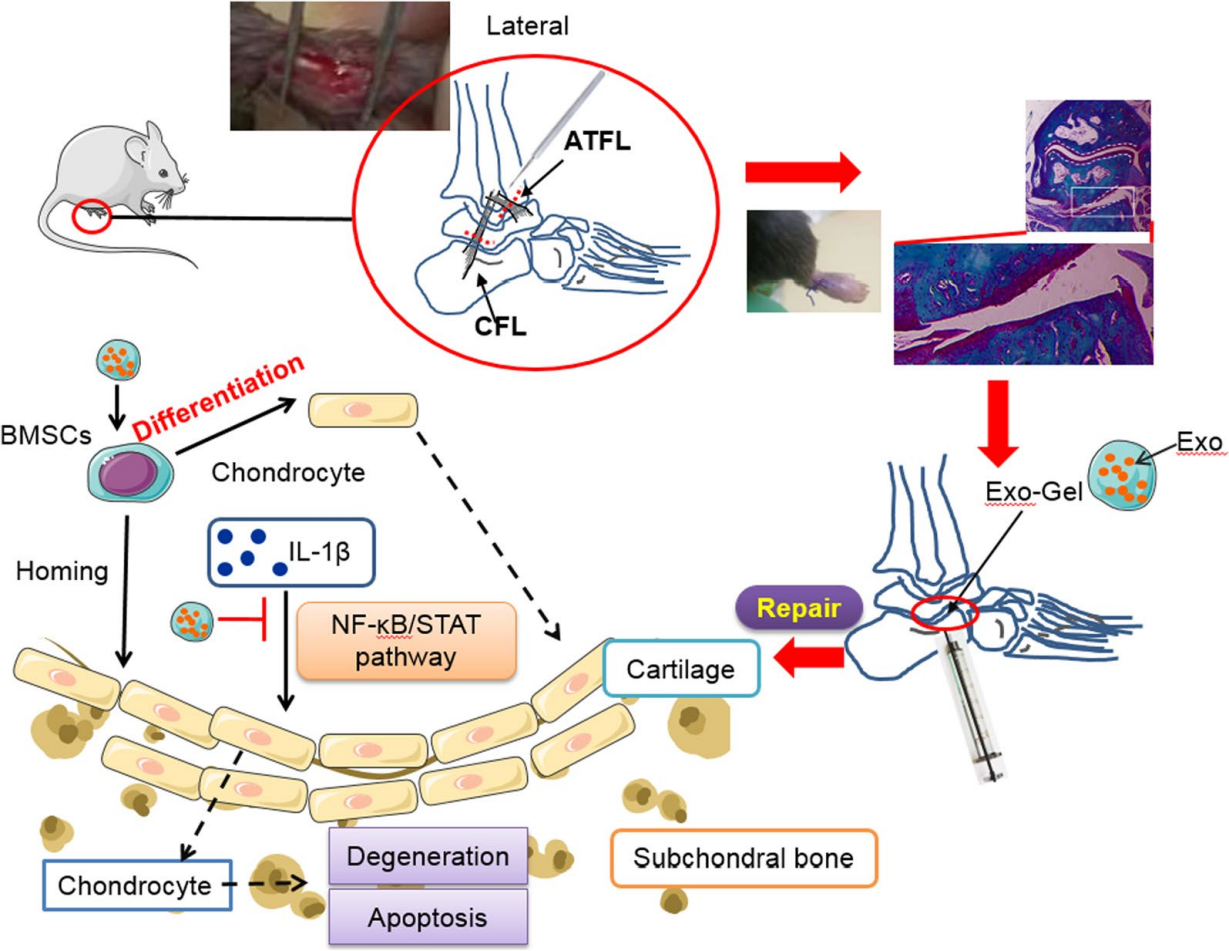

**Supplementary Information:**

The online version contains supplementary material available at 10.1186/s12951-022-01245-8.

## Introduction

Ankle sprain is the most common injury related to physical activity and athletic participation, accounting for about 60% of all injuries in interscholastic sports [1, 2]. The annual medical cost of approximately 2 million acute ankle sprains is estimated to be approximately $ 4.2 billion [3, 4]. Therefore, it is a major public health problem and a major health care burden, although ankle sprain is often regarded as an inconsequential injury. Unfortunately, ankle sprain is not a onetime injury, which is one of the strongest risk factors for recurrent ankle sprain and chronic ankle instability (CAI) [3, 4]. More importantly, as many as 78% of CAI patients develop posttraumatic ankle osteoarthritis (OA) [5, 6]. In addition, lateral ankle sprain is the most common sprain injury, contributed to 13–22% of ankle OA cases and 80% of posttraumatic OA cases [7]. At present, the focus of attention has been on the talocrural joint degeneration caused by lateral ankle sprains. However, due to the similar trauma mechanisms and clinical manifestations of subtalar joint, it leads to underdiagnosis or misdiagnosis of anklesubtalar joint complex injury [8]. To resolve these problems, pathophysiology of ankle OA should be elucidated first. Unlike a major breakthrough in molecular pathophysiological research in knee OA due to the establishment of surgically induced mouse knee OA models in 2005 [9–11], only a few of studies [12, 13] have reported that mechanical instability could be induced in the ankle joint in this mouse model by removal of anterior talofibular ligament (ATFL)/calcaneal fibular ligament (CFL) on its lateral side, to provide a reliable animal model of subtalar OA (STOA). Although such studies substantially promote the development of ankle joint research, the molecular mechanisms of cartilage homeostasis in subtalar joint and intervention for acute lateral ankle sprains are still rarely studied.

Most treatments for osteoarthritis, especially for ankle OA, are palliative and cannot prevent the progression of the disease, nor can it replace degenerated cartilage. At present, there is an urgent need for an effective conservative treatment method that can not only maintain joint function but also relieve pain, so as to improve the quality of life of patients. Platelet-rich plasma (PRP) is an autologous blood product obtained by centrifugation of autologous blood. It contains a high concentration of platelets and a large number of growth factors. Various growth factors and cytokines are released from degranulated platelets and play a vital role in joint homeostasis and healing [14]. In recent years, intraarticular injection of PRP has become a treatment option for knee [15–17] and ankle [18, 19] OA. Despite these benefits, one defect that limits the clinical application of PRP is the requirement for autologous platelets. In addition to these growth factors, stimulation of platelets can also cause them to secrete a large number of extracellular vesicles, including exosomes (Exo, 40–100 nm in diameter) [20]. In the past few decades, the discovery of Exo is one of the most revolutionary contributions to cell biology [21]. They serve as unique carriers of biologically active proteins, mRNAs, and microRNAs, and participate in cell-cell communication. Importantly, recent studies have found that PRP-derived Exo (PRP-Exo) have great potential in the field of tissue repair and regeneration [22, 23], with antiinflammatory effect and positive regulation of cell biological activity [24]. In addition, Exo have the ability to carry large cargo loads to protect the contents, such as transforming growth factor (TGF) β1, platelet-derived growth factor (PDGF) BB, vascular endothelial growth factor (VEGF), stromal cell-derived factor (SDF)-1, also known as CXC-based mutagenic chemokine ligand 12 (CXCL12) [24]. Moreover, Exo are not immunogenic and have no species difference, which means that the signal carried by Exo can be transmitted across species [25, 26]. Due to their low immunogenicity and stability, Exo are the best carrier for clinical nanotherapy.

Considering this immediate washout of Exo, which will lead to a reduction in therapeutic exposure, scholars have conducted indepth studies on the use of injectable hydrogels (Gel) to increase the retention of therapeutic drugs [27–29]. Incorporation of Exo in Gel can allow the controlled release of Exo for a longer period of time, which can even further increase the therapeutic exposure and maximize their efficacy. Injectable Gel based on synthetic materials (such as e.g., poly (ethylene glycol) (PEG) or poly (*N*-isopropylacrylamide)-based)) are easily tunable, and have controllable biochemical properties. Besides, they may be less susceptible to batch-to-batch variations and the most promising candidate systems to increase the local retention of Exo [27, 30]. Previously, a thermoresponsive in situ gel drug delivery system was developed with biosafety poloxamers (Poloxamers 407 and 188) [31], nonionic amphiphilic copolymers composed of a central hydrophobic block of polyoxypropylene (PPO) flanked by two hydrophilic blocks of polyoxyethylene (PEO). Two hydrophilic polyethylene oxides (PEO) [31, 32]. The thermoresponsive property of Gel can be attributed to the phase reverse thermal gelation of the poloxamers under certain condition. However, whether PRP-Exo combined with thermosensitive Gel can prolong Exo retention and enhance therapeutic effects is unknown.

Here, we investigated the use of thermosensitive Gel with poloxamers as a platform for PRP-Exo delivery, aiming to prolong local delivery of Exo to targeted organs. In this study, PRP-Exo incorporated Gel (Exo-Gel) prolonged the release of Exo with biological activity, which promoted the proliferation and migration of mouse bone mesenchymal stem cells (mBMSCs) and chondrocytes, enhanced the chondrogenic differentiation of mBMSCs, and protected chondrocytes aganist apoptosis and degeneration induced by inflammation in vitro. In lateral ankle instability mouse model after transecting ATFL/CFL, compared with Exo alone, Exo-Gel increased the retention of exosomes in the ankle, and therefore improved the therapeutic effect in STOA probably via homing induction of endogenous mBMSCs to the injury site, inducing chondrogenic differentiation, and reducing inflammation-induced cartilage damage.

## Materials and methods

### Reagents and antibodies

The mBMSC culture and differentiation medium was purchased from Cyagen (China). The chondrocyte activator IL-1β was purchased from Sigma-Aldrich (USA). The primary antibodies used in this study included mouse anti-CD9, CD63, CD81, and SOX9 (Santa Cruz Biotechnology, USA); rabbit anti-COL II (Santa Cruze Biotechnology); rabbit anti-PDGFBB, VEGF, SDF-1, COL X, anti-cleaved caspase-3, Bcl-2, Bax, and GAPDH (Abcam, USA); rabbit anti-TGFβ1, Smad2/3, p-Smad2/3, EKR1/2, p-EKR1/2, p38, and p-p38 (Cell Signaling Technology, USA); rabbit anti- COL X and Ki67 (Novus, USA); rabbit anti-Aggrecan, and MMP13 (Proteintech; China).The secondary antibodies used in this study included Alexa-488 conjugated-goat anti-rabbit IgG (H + L) (Jackson ImmunoResearch, USA); horseradishperoxidase–conjugated-goat anti-rabbit IgG (H + L) and horseradish peroxidase–conjugated-goat anti-Mouse IgG (H + L) (Invitrogen, USA); Nuclei was stained with DAPI dihydrochloride (Thermo Fisher Scientific, USA). Flow cytometry anlysis was performed to identify the characterization of mBMSCs stained with FITC-conjugated or PE-conjugated anti-mouse CD44, CD45, CD90, and CD105 (BD, USA).

### Animals

All mice in this study were acquired from Gempharmatech (China). 6–8 weeks old male C57/B6 mice were used for STOA model. 3 w-old mice were used for BMSCs isolation, and newborn pups at postnatal 1 d were used for chondrocytes isolation. All experimental procedures were conducted in conformity with institutional guidelines for the care and use of laboratory animals and protocols, which were approved by the Animal Care and Use Committee of Affiliated Drum Tower Hospital, Medical School of Nanjing University (No. YKK1760).

### Cell culture of mBMSCs

BMSCs were obtained from bone marrow in 3 week-old C57/B6 male mice, and seeded in T25 cell culture flasks with 6 ml BMSC culture medium at 37 ℃. The cells (P0) were passaged when they fused at 80%. These reseeded cells were considered to be the first generation (P1), and so on as the second (P2), third (P3), etc. The BMSC culture medium was refreshed every 3 days, and P3 cells were used in followup experiments.

### Cell culture of chondrocytes

To extract chondrocytes, 1 d-old pups were sacrificed for collection of cartilage from knees. First, cartilage was into small pieces after washing with PBS. Second, the samples were digested in 0.25% trypsin-EDTA (Gibco, USA) solution for 5 min, and DMEM-F12 (Gibco) containing 10% collagenase type II (Sigma-Aldrich) for 6 h at 37 °C, successively. The released chondrocytes were seeded in T25 cell culture flasks. Cells were passaged at a ratio of 1:3 at 80% confluence. The culture medium was refreshed every 3 days.

### Identification of BMSCs

The morphology of P0 and P3 mBMSCs was observed via microscopy (NikonTS2, Japan). Flow cytometry cytoflex (Beckman, USA) was used to detect the surface markers of mBMSCs (CD44, CD45, CD90, and CD105). The data was analyzed by Flowjo 7.6 (TreeStar, USA). Adipogenic, Osteogenic, and chondrogenic differention of mBMSCs were performed according to standard culture methods, and measured via Oil red O staining, Alizarin Red staining, and Alcian blue (AB) staining, respectively. These images were obtained through bright-field microscope (Nikon TS2, Japan).

### Chondrogenic differentiation of mBMSCs

According to the instructions, induction medium (Cyagen, China; containing: TGFβ3 1 mg per 100 ml medium) of chondrogenic differentiation was replaced every 3 days. TGFβ1 neutralizing antibody (1 µg/ml, BD) was administrated in chondrogenic differentiation process. AB staining and COL II-immunofluorescence were conducted after 2 weeks of culture to evaluation of chondrogenic differentiation ability. These results were observed through microscope (Nikon TS2) and Cell imager (Bio-tek Cytation1, USA), respectively.

### PRP extraction

Based on the methods of Tao et al. [23] and Guo et al. [22], whole blood samples were collected from healthy volunteers and placed with acid-citrate Dextrose solution A (ACD-A) in anticoagulant tubes (1 ml ACD-A/9 ml blood). After centrifugation at 160 g for 10 min, platelet-containing plasma was carefully absorbed and transferred to a new centrifuge tube (Beckman coulter, USA) and centrifuged again at 250 g for 15 min. The supernatant plasma was discarded, before the platelet pellet was resuspended in the residual plasma to obtain 4 ml PRP.

### Isolation of PRP-Exo

According to the methods of Torreggiani et al. [20], PRP samples were centrifuged at 250 g for 15 min to obtain PRP microspheres and the platelet pellet was washed with PBS (Ca^2+^-free and Mg^2+^-free, Gibco). After activating 4 ml PRP suspension with 1 ml of 10% CaCl_2_ and 1000 U thrombin (Hunan Yige Pharmaceutical, China), the suspension was centrifuged in series at low speeds (300 g for 10 min, 2000 g for 10 min) to discard cell debris. Then, the supernatant was filtered through a 0.22 μm filter (Millipore, Germany), and the filtrate was transferred to a 15 ml ultrafiltration centrifuge tube (Millipore) under 4000 g centrifugation for 50 min. The liquid was washed with PBS and ultrafiltered at 4000 g again. To purify the Exo, the medium was added onto a 30% sucrose/D_2_O cushion in an Ultra-ClearTM tube (Beckman Coulter, USA) and ultra-centrifuged at 100,000 g for 70 min. After washing by PBS, Exo suspension was ultracentrifuged again at the same high speed for 70 min. The Exo were then carefully resuspended in sterile PBS and stored at − 80 °C for subsequent experiments.

### Identification of PRP-Exo

Nanosight tracking analysis (NTA; Nanosight, UK) was used to measure the concentration and size distribution of Exo. Transmission electron microscopy (TEM; Tecnai 12, Philips, The Netherlands) was used to identify the morphology of Exo after incorporated in Gel. The biosignature proteins of PRP-Exo, consisting of CD81, CD9, CD63, Tgfβ1, PDGFBB, VEGF, and SDF-1 were determined by western blotting assays.

### Gel and Exo-Gel preparation

The production methods of Gel have been described in our previous report [33], the total polymer concentration of blank Gel was set to 22.9% (w/w), of which the concentration of Poloxamer 407 (PEO_101_-PPO_66_-PEO_101_, Sigma-Aldrich) and 188 (PEO_80_-PPO_27_-PEO_80_, Aladdin, China) was fixed at 17.9% (w/w) and 5% (w/w), respectively. Poloxamer 407 and 188 were added into ddH_2_O according to the ratio to stir and mix evenly at 4 ºC. Then, the Gel in liquid state was fully mixed with extracted Exo, and this Exo incorporated Gel (Exo-Gel) system was freeze-dried by lyophilizer (Christ Alpha1-2, Germany). After that, it was further detected by SEM (Philips). Liquid Gel was transferred to transwell inserts with 8 μm pore size (Millipore) and solidifed by raising temperature to 37.0 ℃ in the cell incubator. The conditioned medium from Exo-Gel (200 µg Exo incorporated in 100 µl thermossensitive Gel) incubation for 1 week was obtained by placing the transwell inserts in wells containing 1 ml of medium, and used to coculture with mBMSCs and chondrocytes for multiple functional tests, such as proliferation, migration, differentiation, and apoptosis.

### Thermoresponsive release profile of Exo-Gel

For release studies, the same amount of Exo-Gel was placed into transwell inserts as described above and incubated in medium at 25 and 37 °C. The supernatant was removed and fresh medium was added at different time-points. The number of Exo released into the medium was determined using the Bradford Protein Assay Kit (Beyotime, China) and the size distribution of Exo at 2d postincubation was detected by NTA (Nanosight).

### Uptake of Exo by mBMSCs and chondrocytes

For the fluorescent labeling of Exo, DiR-solution (Eugene, USA) was added to PBS and incubated as manufacturer instructed, then centrifuged 100,000 g at 4 °C for 1 h to remove excessed dye. These DiR-labeled Exo incorporated Gel (200 µg/100 µl) placed in a transwell insert and and cocultured with mBMSCs and chondrocytes in the bottom well for 48 h. Cells were washed with PBS thrice, fixed in 4% paraformaldehyde (PFA), and permeabilized with 0.05% Triton X-100 for 5 min, followed by incubation with FITC Phalloidine (Yeasen, China). Nuclei was stained with DAPI dihydrochloride. Fluorescent images were photographed by Cell imager (Bio-tek Cytation1).

### Cell Counting Kit-8 analysis

Viability of mBMSCs and chondrocytes was evaluated with a Cell Counting Kit-8 (CCK-8) assay (Dojindo, Japan). After 0, 12 h, 1 d, and 2 d, the wells were washed with PBS thrice, and CCK8 solution (10 µl; 1:10 dilution) in fresh culture medium was added to wells and incubated for 2 h at 37 °C. The absorbance was measured at 450 nm with a microplate reader (ELx800, Bio-tek). The cell growth curve is drawn according to the measured OD value.

### 5-Ethynyl-2’-deoxyuridine (EDU) analysis

Cell proliferation was evaluated using an EDU kit (RiboBio, China) in accordance with the manufacturer’s protocol. Briefly, mBMSCs and chondrocytes were seeded into 24-well plates 20, 000 per well and incubated with EDU medium for 2 h. After washing with PBS, the cells were fixed in 4% PFA and permeabilized with 0.05% Triton X-100 for 5 min. At last, we added staining solution to each well followed by incubating at room temperature for 30 min. Nuclei was stained by Hoechst 33,342 and the images were taken under Cell imager (Bio-tek Cytation1).

### Migration assay

Transwell assay was used to analyze the migration ability of BMSCs and chondrocytes under different treatments. Briefly, 20,000 cells were seeded into the upper chamber of a 24-well transwell plate (Millipore; pore size: 8 μm) after which 600 µL/well medium treated differently were added to the lower chamber. Following incubation for 24 h, cells that migrated to the lower surface of the filter membrane were stained with 0.5% Crystal Violet for 30 min and observed by microscopy (Nikon TS2). Migratory activity was assessed by counting the number of cells.

A scratch wound assay was also performed to evaluate cell migration capability. In brief, cells were cultured in 6-well plates to 100% confluence. We used a sterile 200 µl pipette tip to scrape the cell layer. After washing with PBS, the images of each processing groups were recorded at 0 and 24 h after scratching by microscopy (Nikon TS2).

### Real-time quantitative PCR

Total RNA was extracted from the mBMSCs using the RNA-Quick Purification Kit (Vazyme, China) and the cDNA was amplified using the HiScript II Q RT SuperMix for qPCR (Vazyme) according to the manufacturer’s instructions. The qPCR was performed with SYBR Green PCR Master Mix (Vazyme) on using ABI steponeplus real-time PCR system (Applied Biosystems, USA). The level of expression was standardized to GAPDH, and the relative expression level was evaluated using the 2^−ΔΔCT^ approach. The primers used in this experiment were synthesized by GENEbay (China) with the following sequence: GAPDH (Forward: 5′-TCATGGGTGTGAACC ATGAGAA-3′, Reverse: 5′-GGCATGGACTGT GGTCATG AG-3′), COL II (Forward: 5′-CACACTGGTAAGTGGGGCAAGACCG-3′, Reverse: 5′-GGATTGTGTTGTTTC AGGGTTCGGG-3′), ACAN (Forward: 5′-CCTGCTACTTCATCGACCCC-3′, Reverse: 5′-AGATGCTGTTGACTCGAACCT-3′), and SOX9 (Forward: 5′-GGTGC T CAAGGGCTACGACT-3′, Reverse: 5′-GGGTGGTCTTTCTTGTGCTG-3′).

### Chondrocyte apoptosis by flow cytometry

Chondrocyte apoptosis were assessed using an Annexin V-FITC/PIapoptosis detection kit (Keygen Biotech, China). According to the manufacturer’s protocol, the chondrocytes were digested by 0.25% trypsin without EDTA, washed with PBS and centrifuged at 300 g for 5 min. The collected cells (500,000/per tube) were resuspended in binding buffer (500 µl) containing 5 µl Annexin V-FITC and 5 µl Propidium Iodide (PI) after 10 min incubation in dark and detected by flow cytometer (Beckman, USA).

### Cell fluorescence analysis

Cells were fixed with 4% PFA for 20 min, washed with PBS three times, permeabilized with 0.05% Triton X-100 for 10 min and blocked with 10% goat serum for 2 h. Then, samples were incubated with primary antibodies overnight at 4 °C, followed by Alexa-488 conjugated-goat secondary antibody (Jackson ImmunoResearch) for 2 h at room temperature. After triple washing by PBS, nuclei were stained with DAPI and fluorescent images were acquired using fluorescence microscope (Leica DMI3000B, Germany) and Cell imager (Bio-tek Cytation1).

### Western blot assay

Total protein was purified from cells by RIPA lysis (keygen Biotech, China). Protein concentration was quantified by Bradford Protein Assay Kit (Beyotime). Equal amounts of protein were separated by SDS-PAGE, and transferred to PVDF membranes (Millipore). Subsequently, PVDF membrane was blocked by 5% bovine serum albumin for 2 h at room temperature prior to incubating overnight at 4 °C in respective primary antibodies. Membranes were then incubated for 2 h at room temperature with the appropriate secondary antibodies. The bands were exposed by applying chemiluminescence kit (Beyotime) in an imaging system (Bio-Rad, USA). Quantification of band intensity was also performed by ImageJ (National Institutes of Health, USA).

### Animal experiments

C57 male mice aged 6–8 weeks were used to performed chronic ankle instability model by transecting lateral ligaments (ATFL/CFL) as previously described [12, 13]. Under anesthetic conditions, CFL, connecting from the apex of the fibular malleolus to the lateral surface of the calcaneus, and ATFL, running from the distal anterior tip of the fibula to the lateral talar neck were both excised at its attachment sites. The lateral ankle capsule, which connects from anterior aspects of fibula to the lateral side of the talus, was incised after removal of CFL and ATFL. After surgery, 2 µl PBS, 2 µl blank Gel, 2 µl PBS containing 2 µg Exo, or 2 µl Exo-Gel (4 µg Exo/2 µl Gel) were respectively injected into subtalar joint, and consequently depending on the composition of the injection, the mice were assigned to below groups: PBS, Gel, Exo, or Exo-Gel (n = 6/group). A sham operation was performed on the opposite ankle using the same skin incision without ligament/tendon resection. To identify sustained-release of Gel, DiR-labeled Exo (4 µg/2 µl PBS) and Exo-Gel (4 µg/2 µl) were locally injected into subtalar joints via precooled Hamilton precision syringe (Hamilton Co., Ltd, Switzerland), respectively, and the IVIS Spectrum imaging system (PerkinElmer, USA) was used to observe right subtalar joints on 3, 7, 14, and 28 d after surgery. A sham operation was performed on the opposite ankle using the same skin incision without ligament/tendon resection. The entire operation was finished after the suture of the incision. Mice were allowed to move freely in the cages and had free access to food and water. The mice were sacrificed at 4 and 8 weeks after the surgery.

### Histomorphological detection

Ankle samples at 8 weeks postinjury were fixed in 4% PFA, decalcified in EDTA, and embedded in paraffin. Five-µm frontal sections were prepared and stained with safranin O and fast green, HE, toluidine blue (TB), and alcian blue (AB), respectively, according to the standard protocol, and the morphology of the tissue was observed by microscopy (Nikon TS2, Japan).

#### Immunohistochemical analysis

Ankle samples at 4 weeks postinjury were prepared according to standard dewaxing procedure. The slices were soaked in citric acid buffer (10 mM citric acid, pH 6.0) at 100 °C for 10 min to display antigens and 3% hydrogen peroxide was added for 10 min to inactivate endogenous peroxidase. Then, immunohistochemical staining was performed using a previously reported protocol. After incubation with the primary antibodies at 4 °C overnigh, the slides were subsequently stained with horseradish peroxidase-conjugated secondary antibody (Invitrogen). The presence of antigen in cartilage was determined by counting the number of positively stained chondrocytes by microscopy (Nikon TS2).

### TUNEL staining

TUNEL (Roche, Switzerland) staining of cells and tissue slides was performed in dark for 30 min at 37 °C based on the manufacturer’s protocol. Nuclei was stained with DAPI. Images were captured by using Cell imager (Bio-tek Cytation1).

### Statistical analysis

GraphPad 7.0 and SPSS 19.0 software were used to process and analyze the data. All independent experiments were performed in triplicate and all quantitative data were expressed means ± SEM. Data were analyzed using the Student’s T-test or one-way or two-way ANOVA to determine statistical differences. A value of p < 0.05 was considered as statistically significant.

## Results

### Characterization of PRP-Exo

**Fig. 1:**
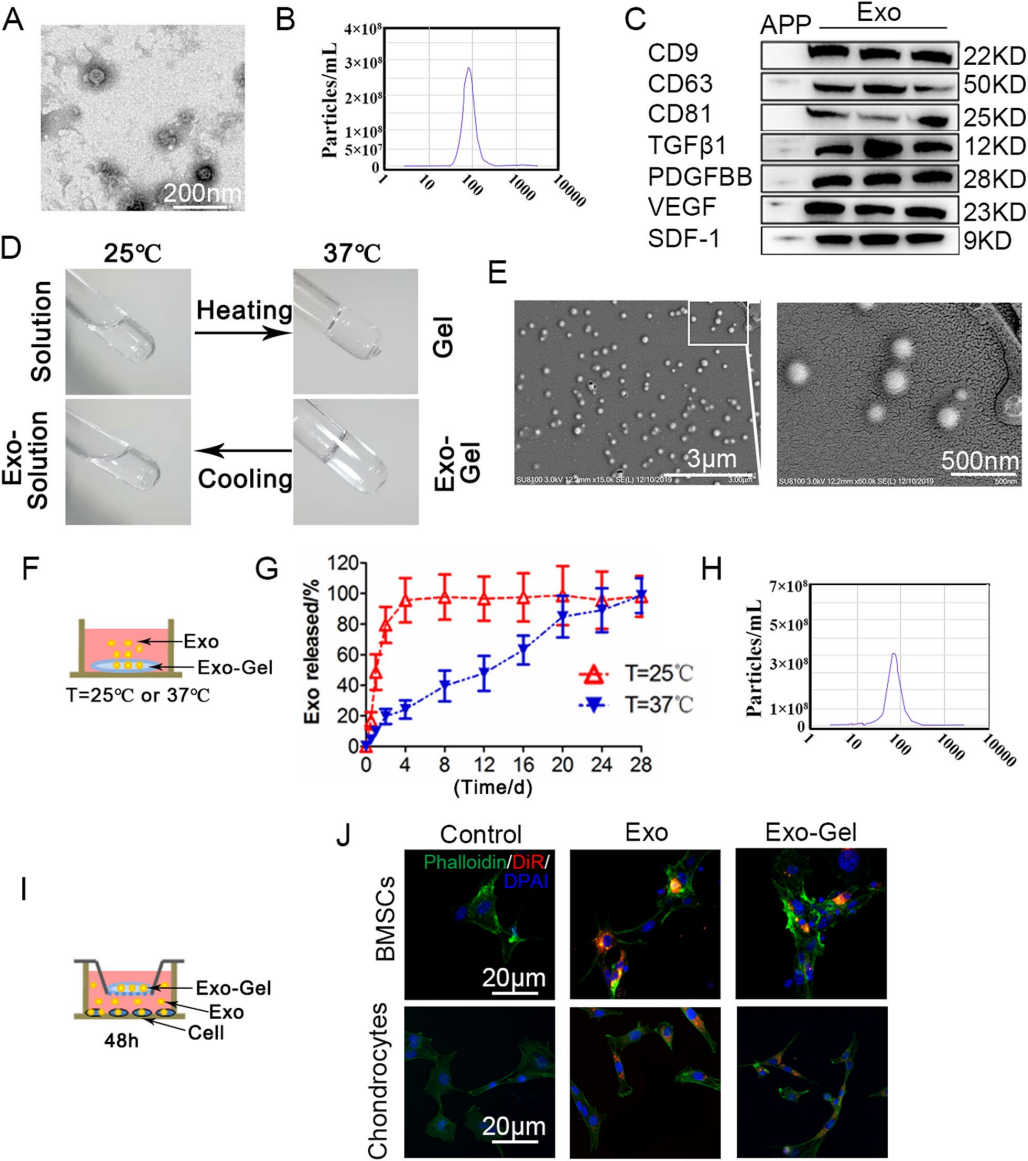
Characterization of PRP-Exo and Exo-Gel. **A** Morphology of Exo examined by TEM. Scale bar: 200 nm. **B** Particle size distribution of Exo measured by NTA. **C** Western blotting analysis of the surface biomarkers CD9, CD63, and CD81 on Exo, and the encapsulated proteins TGFβ1, PDGFBB, VEGF, and SDF-1, as compared with APP. **D **Photographs of Exo-Gel demonstrated the thermoresponsiveness of the formulation, for which the polymer solution at room temperature (25 °C) reversibly transformed into a hydrogel at 37 °C. **E** SEM of the morphology of Exo loaded in Gel (bar = 500 nm). **F** Exo release profles from Gel at 25 and 37 °C. **G** Thermoresponsive release profile of Exo loaded in Gel detected by Bradford Protein Assay at different time-points. Error bars represent the means ± SEM (n = 3). **H** Particle size distribution of Exo released from Gel measured by NTA. **I** In vitro setup to determine uptake of DiR-labeled Exo released from Gel at 48 h by mBMSCs and chondrocytes. **J** Fluorescent images of mBMSCs and chondrocytes cocultured with Exo-Gel showing that Exo could be taken up by mBMSCs and chondrocytes after release from Gel

In order to fully characterize the purified PRP nanoparticles, transmission electron microscopy (TEM), nano visual tracking analysis (NTA) and western blotting were used. TEM shows that the diameter of round nanoparticles is between 40 and 100 nm, which is consistent with the literature report [20] (Fig. 1A). The particle size distribution of NTA is similar (Fig. 1B), indicating the existence of Exo. In addition, western blotting (Fig. 1C) showed that PRP-Exo-specific exosomal surface markers CD9, CD63, and CD81 were positive, further confirming their identity. At the same time, the Exo cargo was analyzed, and it was found that PRP-Exo contained TGFβ1, PDGFBB, VEGF and SDF-1. Since the main bioactive molecules in platelet pellet (PP), are encapsulated in the Exo [20], and released after activation, the surface markers of Exo and growth factors in the activated platelet pellet (APP) barely existed as compared with PRP-Exo (Fig. 1C).

#### Characterization of Exo-Gel

As previously reported by our team [33], the thermossensitive Gel was an ideal injectable carrier to deliver drugs, and the total polymer concentration of blank hydrogel was set to 22.9% (w/w), of which the concentration of Poloxamer 407 and 188 was fixed at 17.9% (w/w) and 5% (w/w) respectively. When Exo were mixed with Gel, Exo-Gel still was reversibly converted from a liquid polymer solution at room temperature into a hydrogel at 37 °C as blank gel, showing a thermoresponsiveness, which may highlight that the presence of Exo does not hamper Gel gelation process (Fig. 1D). The morphology of Exo loaded in Gel was examined by scanning electron microscopy (SEM) and the outline of round nanoparticles could be clearly observed (Fig. 1E). To study the use of thermossensitive Gel for sustained Exo delivery, 100 µl of Gel with 200 µg of Exo transferred to an insert of a transwell system containing medium in the bottom compartment at 25 and 37 °C (Fig. 1F), and conditioned medium was sampled at several time-points and quantified using the Bradford Protein Assay Kit (Fig. 1G). In the case of Exo-Gel, the vast majority of Exo released to the medium after 1 day at 25 °C. After increasing the temperature to 37 °C, Exo continually released from Gel to the medium, lasting for about 1 month. Additionally, NTA analysis showed that the particle size distribution of Exo released from Gel in the conditioned medium sampled at 2d was similar with initial PRP-Exo (Fig. 1H). Importantly, Exo have the ability to transfer their biological cargo, including proteins and RNA, between cells after Exo uptake by the recipient cell [34], which is key for biologically active of Exo. Therefore, to ensure that Exo maintain their integrity after release from Gel, we assessed Exo uptake by mouse bone marrow stromal cells (mBMSCs) and chondrocytes. Then, mBMSC phenotype was identified using morphological images (Additional file 1: Fig. S1A, B), flow cytometry (Additional file 1: Fig. S1C--F) and three-lineage differentiation (Additional file 1: Fig. 1SG–I). DiR-labeled incorporated Gel placed in a transwell insert and cocultured with mBMSCs and chondrocytes in the bottom well (Fig. 1I). After 48 h, DiR-labeled Exo were visible in mBMSCs or chondrocytes cocultured with Gel (Fig. 1J). These results indicated that Gel matrix had the ability to sustain the release of Exo without characteristic change for a long period of time at the body temperature.

### Exo-Gel promoted proliferation and migration of mBMSCs

**Fig. 2:**
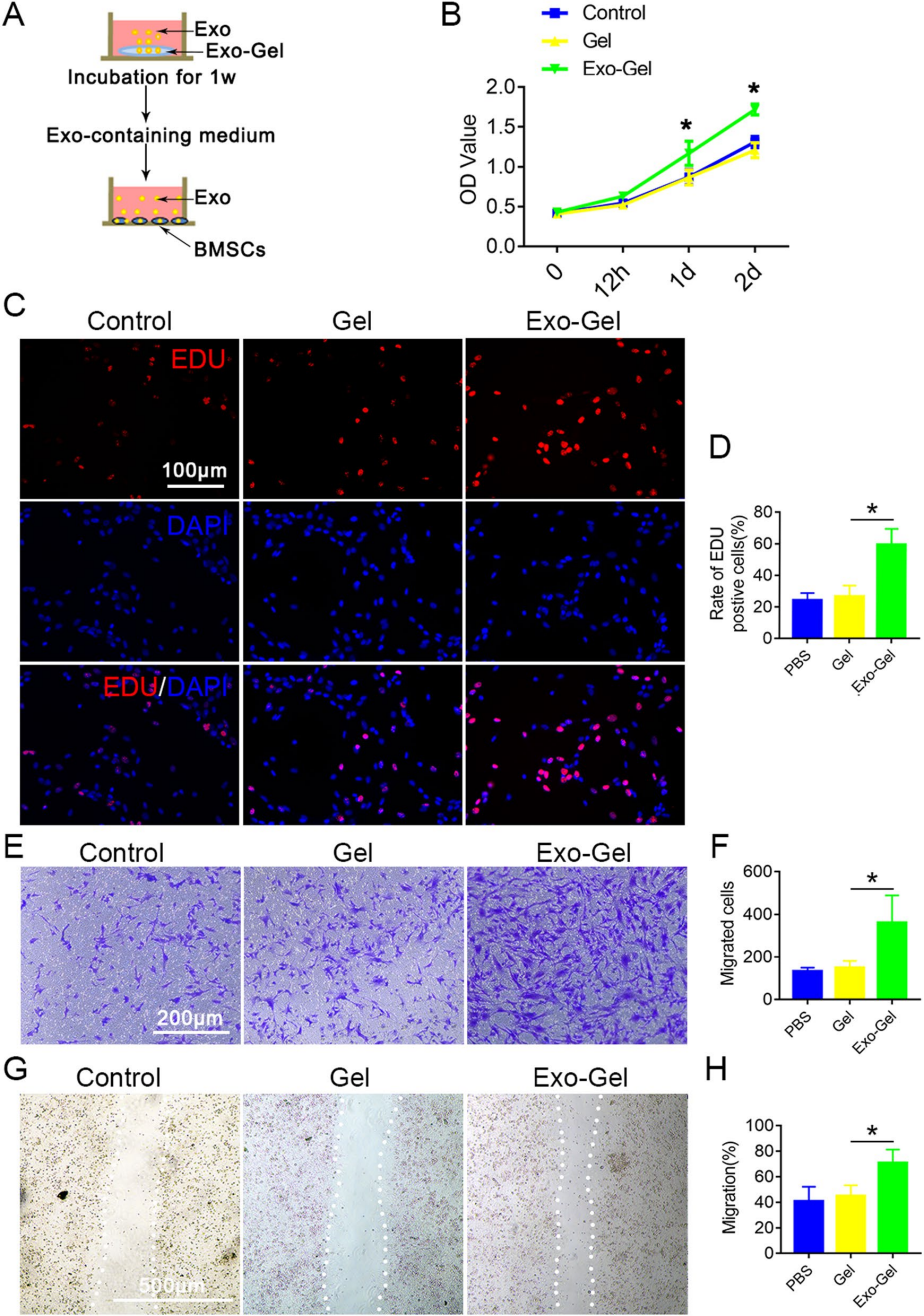
Exo-Gel promoted proliferation and migration of mBMSCs in vitro. **A** In vitro setup to determine if Exo released from Exo-Gel maintain their ability in proliferation and migration of mBMSCs. **B** The cell viability of mBMSCs cocultured with conditioned medium examined by CCK-8 analysis. Data are expressed as means ± SEM (n = 6). *p < 0.05, Exo-Gel group compared to Gel group. **C** Proliferation of mBMSCs was confirmed by EDU kit assay (bar = 100 μm). **D** Quantification of rate of EDU positive cells in **C** was performed. Data are expressed as means ± SEM (n = 8). (E) Transwell assay to show a representative image of migrating mBMSCs (bar = 200 μm). **F** Quantitative analysis of migrating cells in **E**. Data are expressed as means ± SEM (n = 6). **G** A representative image showing the migration ability of mBMSCs by the scratch test (bar = 500 μm). (H) Quantitative analysis of mBMSC migration rate (**G**). Data are expressed as means ± SEM (n = 6). *p < 0.05

BMSCs have the ability to differentiate into chondrocytes and are a well-known cell source for cartilage tissue engineering [35, 36]. Therefore, the key step is to stimulate the chemotaxis of a sufficient number of endogenous BMSCs to the injured site. SDF-1 is abundant in PRP [37], and the analysis of Exo protein content revealed a higher amount of SDF-1 as compared to APP (Fig. 1C). Binding to CXCR4 on BMSCs, SDF-1 can drive cell migration in tissue repair after injury [38, 39] or cartilage repair after osteoarthritis [40, 41]. It has also been shown to induce the migration of BMSCs to other locations, such as the brain [42] and heart [43]. Therefore, we believed that SDF-1 released by Exo induced endogenous BMSCs recruitment and promoted the repair of OA. Initially, we evaluated if Exo released from Exo-Gel after 1 week maintained their functional ability in targeted mBMSCs, mBMSCs were cultured in conditioned medium supplemented with Exo-Gel for a series of functional assays (Fig. 2A). The proliferation of cells was examined by CCK-8 analysis (Fig. 2B) and confirmed by EDU assay (Fig. 2C). The results revealed that conditioned medium after Exo-Gel incubation markedly increased proliferative capability of cells. Meanwhile, the conditioned medium could promote migration of mBMSCs evaluated using the transwell and scratch wound assays. These results showed that Exo released by Exo-Gel after incubation for 1 week in vitro still had biological activity and could promote the proliferation and migration of mBMSCs.

### Exo-Gel promoted chondrogenic differentiation of mBMSCs

**Fig. 3:**
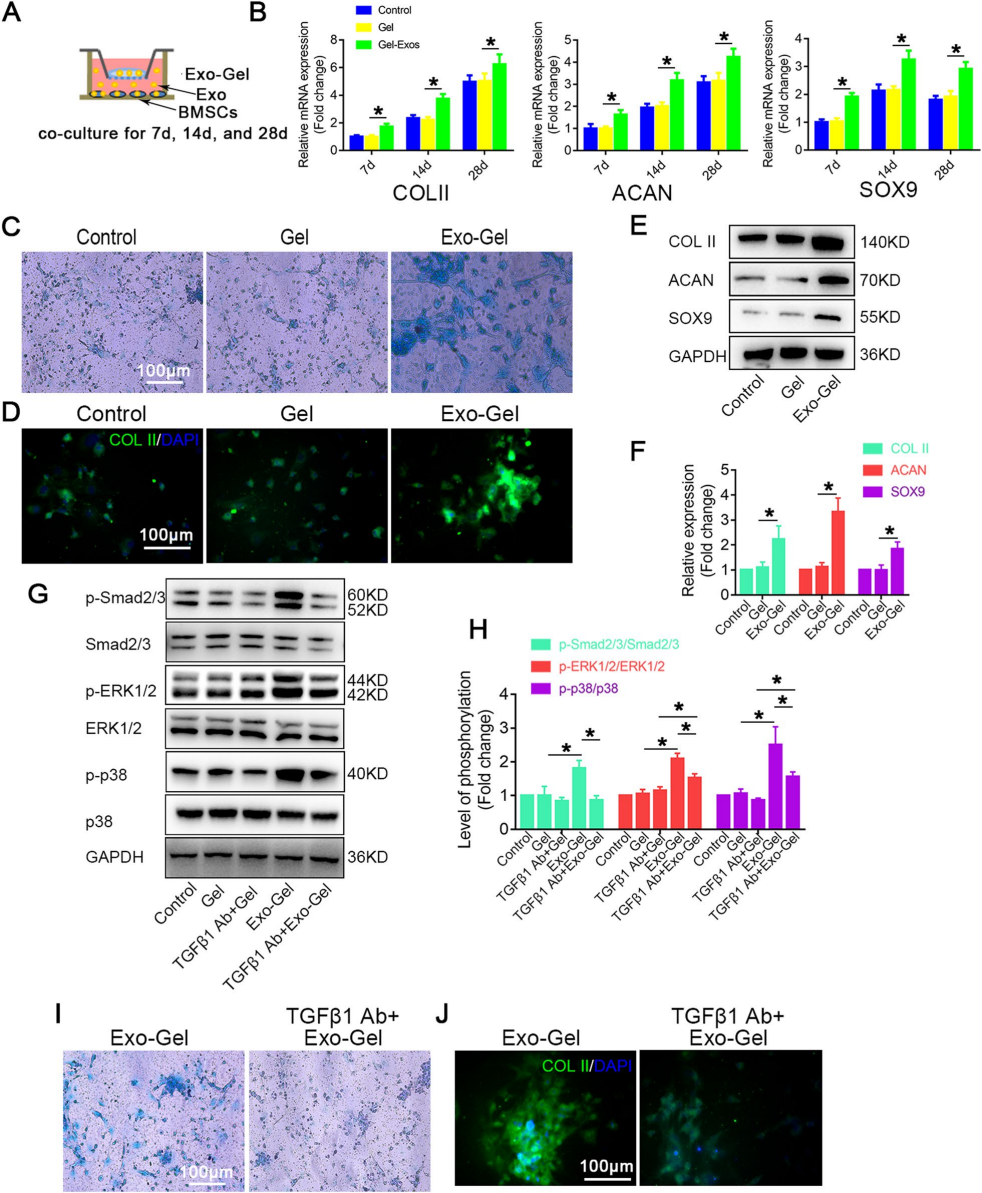
Exo-Gel enhanced chondrogenic differentiation mBMSCs in vitro. **A** In vitro estimate the ability of Exo-Gel by coculture in chondrogenic differentiation of mBMSCs. **B** RT-PCR analysis for COL II, ACAN and SOX9 mRNA in mBMSCs treated as described in (A) at different time-points. Data are represented as means ± SEM (n = 3). **C** AB staining of mBMSCs in different treatment groups under light microscopy on the 14th day of induction of differentiation (bar = 100 μm). **D** Immunofluorescent assay of mBMSCs for COL II (red) and DAPI (Blue) in different treatment groups at the same time-point in **C** (bar = 100 μm). **E** Western blot assay to detect the protein expression levels of COL II, ACAN and SOX9 at the same time-point in **C**. **F** Quantification of protein expression in **E**. GAPDH was used as loading control. Data are expressed as means ± SEM (n = 3). **G** Representative Western blot showing the levels of total protein and phosphorylated protein for the indicated molecules (Smad2/3, ERK1/2, and p38) in mBMSCs with or without TGFβ1 neutralizing antibody at the same time-point in **C**. **H** Quantification of protein expression and the level of signaling activation in **G**. GAPDH was used as loading control. Data are expressed as means ± SEM (n = 3). **I** AB staining of mBMSCs with or without TGFβ1 neutralizing antibody under light microscopy on the 14th day of differentiation (bar = 100 μm). **J** Immunofluorescent assay of mBMSCs for COL II (red) and DAPI (blue) under the same condition as (**I**) (bar = 100 μm). *p < 0.05

Previous study has showed a higher amount of growth factors in PRP-Exo [20], which play an important role in chondrogenic differentiation. Subsequently, Exo-Gel was cocultured with mBMSCs and chondrogenic differentiation at different time-points of induction culture was evaluated by RT-PCR (Fig. 3A). Our results indicated that mRNA expressions of type II Collagen (COL II), Aggrecan (ACAN), and SOX9 representing chondrogenic differentiation from the 7th day after coculture with Exo-Gel were significantly higher than those in blank Gel group (Fig. 3B). Alcian blue (AB) staining (Fig. 3C) and immunofluorescence staining (Fig. 3D) confirmed that cartilage clumps in Exo-Gel group on the 14th day of induced differentiation were more obvious. At the same time-point, the protein expressions of COL II, ACAN, and SOX9 were also showed a significant increase in Exo-Gel group as compared to Gel group (Fig. 3E, F).

**Fig. 4:**
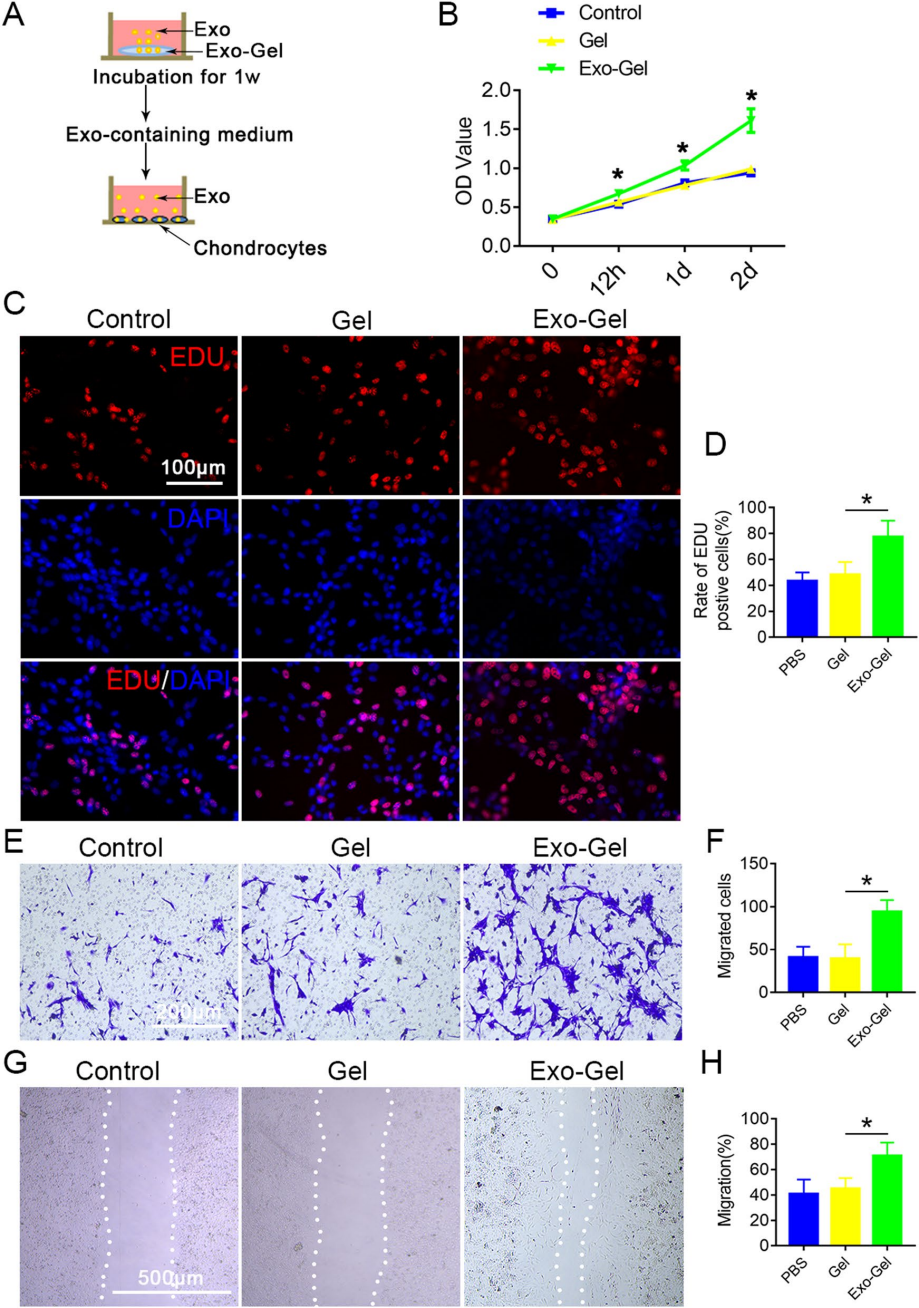
Exo-Gel promoted proliferation and migration of chondrocytes in vitro. **A** In vitro investigate the effect of conditioned medium after Exo-Gel incubation for 1 week on proliferation and migration of chondrocytes by co-culture. **B** The cell viability of chondrocytes examined by CCK-8 analysis. Data are expressed as means ± SEM (n = 6). *p < 0.05, Exo-Gel compared to Gel group. **C** Proliferation of chondrocytes was confirmed by EDU kit assay (bar = 100 μm). **D** Quantification of rate of EDU positive cells in **C** was performed. Data are expressed as means ± SEM (n = 8). (E) Transwell assay to show a representative image of migrating chondrocytes (bar = 200 μm). **F** Quantitative analysis of migrating cells in **E**. Data are expressed as means ± SEM (n = 6). **G** A representative image showing the migration ability of chondrocytes by the scratch test (bar = 500 μm). **H** Quantitative analysis of chondrocyte migration rate (**G**). Data are expressed as means ± SEM (n = 6). *p< 0.05

As a key factor in the regulation of chondrogenic differentiation, TGFβ isoforms, including TGFβ1, TGFβ2 and TGFβ3, activates Smad2/3, ERK1/2 and p38 signaling pathways during the process [44], which is consistent with our results (Fig. 4G, H). In this study, TGFβ3 was applied to induce chondrogenic differentiation, and our result confirmed that Exo contained a large amount of TGFβ1 protein (Fig. 1C).To verify whether Exo enhanced chondrogenic differentiation ability through the content of TGFβ1, TGFβ1 neutralizing antibody was administrated in Exo-Gel and BMSCs co-culture system (Fig. 3G, H). After treatment with TGFβ1 (1 µg/ml) neutralizing antibody, Smad2/3, ERK1/2 and p38 phosphorylation did not change significantly. As we expected, in Exo-Gel group, it could be observed that TGFβ1 neutralizing antibody significantly downregulated activation of Smad2/3, ERK1/2 and p38 signals and significantly inhibited the formation of cartilage (Fig. 3I, J). These results suggested that Exo increased chondrogenic differentiation ability of mBMSCs by releasing TGFβ1. Interestingly, after administration of TGFβ1 neutralizing antibody in Exo-gel group and Gel group, activation level of ERK1/2 and p38 signal in the former was still higher than that in the latter (Fig. 3G, H), which hinted that other growth factor, such as fibroblast growth factor (FGF) [45–47] in PRP-Exo released from Exo-Gel assisted regulation of chondrogenic differentiation.

### Exo-Gel promoted proliferation and migration of chondrocytes

In order to further determine the potential therapeutic effect of Exo-Gel on cartilage repair, we evaluated the growth of chondrocytes by in vitro CCK8 test and EDU analysis (Fig. 4A–D). The effect of Exo-Gel on the increasing proliferation of chondrocytes is similar to that of mBMSCs. Transwell and scratch tests showed that Exo-containing culture after Exo-Gel incubation could markedly enhance the migration ability of chondrocytes (Fig. 4E–H). In general, these findings indicated that compared with PBS and Gel treatment, Exo-Gel could promote the proliferation and migration to a greater extent in vitro.

### Exo-Gel inhibited IL-1β-induced chondrocyte apoptosis and degeneration

**Fig. 5:**
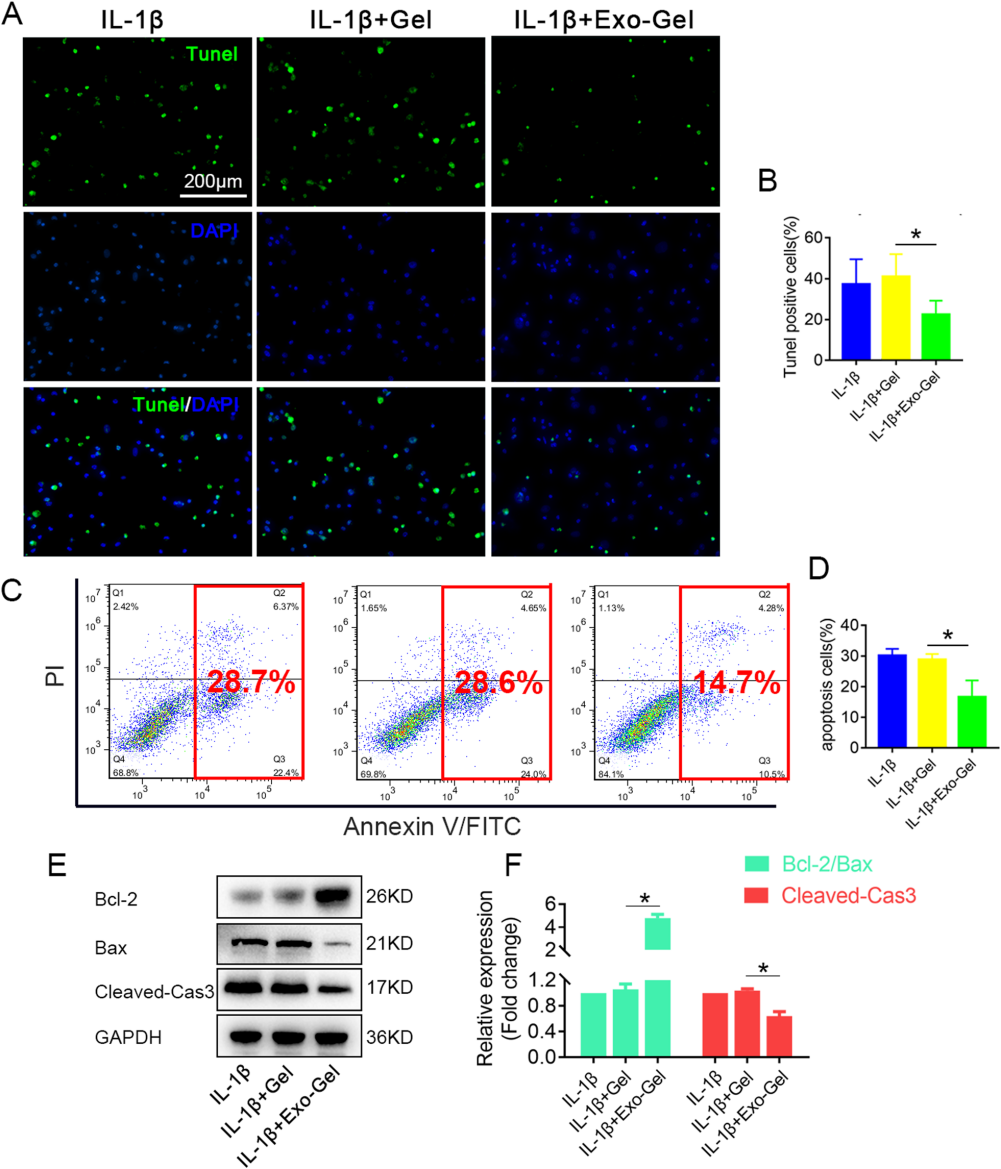
Exo-Gel suppressed IL-1β-triggered chondrocyte apoptosis in vitro. **A** In vitro investigate the effect of Exo-Gel on apoptosis of chondrocytes treated with IL-1β (10 ng/ml) for 24 h. TUNEL (green) staining to analyze chondrocyte apoptosis, DAPI (blue) staining for cell nuclei (bar = 200 μm). **B** Quantitative assessment of the proportion of apoptotic cells in **A**. Data are expressed as means ± SEM (n = 6). **C** Annexin V/FITC/PI double-staining flow cytometry to detect the effect of Exo-Gel coincubated medium on IL-1β-triggered chondrocyte apoptosis. **D** Quantification of apoptotic cells (Annexin V-positive) in **C**. Data are expressed as means ± SEM (n = 4). **E** Representative Western blots of cleaved-Cas3, Bcl-2, and Bax expression in chondrocytes in different groups. **F** Quantification of cleaved-Cas3 protein expression and the ratio of Bcl-2 to Bax in chondrocyte in **E**. GAPDH was used as loading control. Data are expressed as means ± SEM (n = 3). *p < 0.05

Among the cytokines believed to play a role in progression of OA, IL-1β and TNF-α are known to be the predominant mediators of inflammation [48–50], which aggravate the catabolic imbalance in OA cartilage and exacerbate degradation and alterations of articular cartilage matrix. Therefore, we evaluated the protective effect of conditioned medium after incubation with Exo-Gel for 1 week on chondrocytes in the presence of IL-1β (10 ng/ml) in vitro. Firstly, we observed the influence of Exo-Gel on the cell apoptosis of chondrocytes by TUNEL staining and Annexin V-FITC/PI flow cytometry (Fig. 5A–D). Conditioned medium after Exo-Gel incubation remarkably decreased the rate of apoptosis of chondrocytes triggered by IL-1β. Western blotting (Fig. 5E, F) demonstrated that the expression of cleaved-caspase3 (Cleaved-Cas3) protein in chondrocytes in response to IL-1β treatment was higher than that in the cells cocultured with conditioned medium from Exo-Gel incubation. Meanwhile, the Bcl-2/Bax ratio calculated from Western blotting result was significantly increased in the IL-1β + Exo-Gel group. These results indicated that Exo-Gel could attenuate inflammatory apoptosis during cartilage degeneration.

**Fig. 6:**
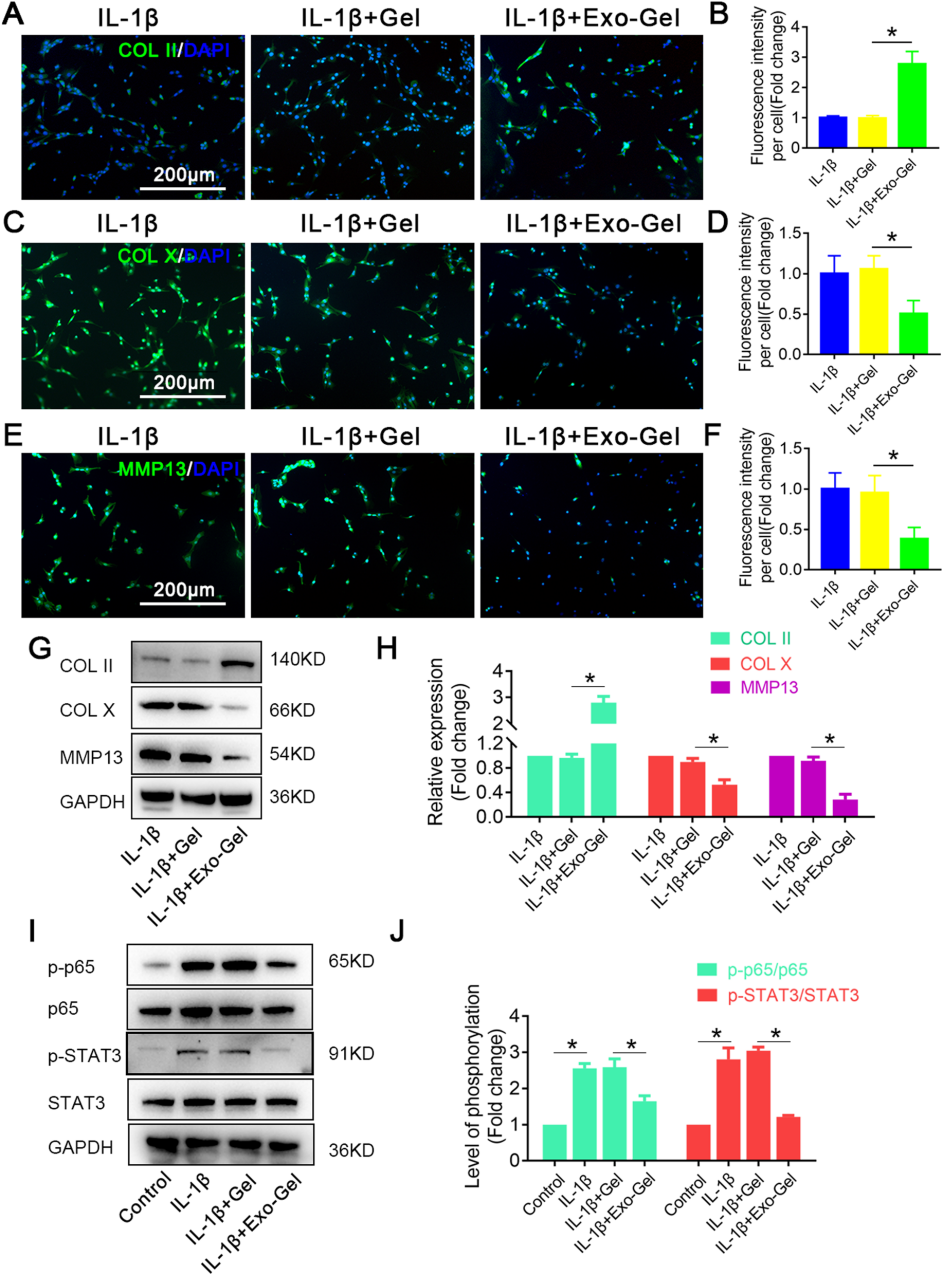
Exo-Gel suppressed IL-1β-triggered chondrocyte degeneration and enhanced anabolism in vitro. **A** Immunofluorescent assay to detect the expression of COL II (Gree), COL X (Green), and MMP13 (Green) in chondrocytes in different treatment groups, DAPI (blue) staining for cell nuclei (bar = 200 μm). **B** Quantification of COL II, COL X, and MMP13 fluorescence intensity in **A**. Data are expressed as means ± SEM (n = 6). **C** Representative Western blots of COL II, COL X, and MMP13 expression in chondrocytes in different groups. **D** Quantification of COL II, COL X, and MMP13 protein expression in chondrocytes in **C**. GAPDH was used as loading control. Data are expressed as means ± SEM (n = 3). **E** Representative Western blot showing the levels of total protein and phosphorylated protein for the indicated molecules (p65 and STAT3) in chondrocytes in different treatment groups. **F** Quantification of protein expression and the level of signaling activation in **E**. GAPDH was used as loading control. Data are expressed as means ± SEM (n = 3) *p < 0.05

Furthermore, inflammatory cytokines affect the physiological metabolism of chondrocytes by activating catabolic pathways, and promote the imbalance of catabolic and anabolic balance. Matrix metallopeptidase (MMP) 13 is a catabolism-related factor and widely known as a proteolytic enzyme, that can cause the degradation of extracellular matrix (ECM) components, especially COL II and ACAN [51], which is strongly related to the pathological process of OA. Moreover, transcription factor nuclear factor kappa B (NF-κB), activated by IL-1β in chondrocytes, shuts down almost all anabolic pathways, including the production of COL II and ACAN [49, 52, 53]. Single cell RNA-seq studies in OA, have shown that chondrocytes undergo phenotypic changes and become hypertrophy, leading to cartilage damage and aggravating diseases [54–56]. In addition, hypertrophic chondrocytes produce alkaline phosphatase, leading to matrix calcification and increase the expression of hypertrophic-related gene, type X Collagen (COL X) [57]. MMP13 as other main characteristic is also upregulated in hypertrophic chondrocytes [55, 56]. Then, we analyzed the protein expression of COL II, COL X and MMP13 in IL-1β-stimulated chondrocytes cocultured with conditioned medium after Gel or Exo-Gel incubation for 24 h. Immunofluorescence analysis (Fig. 6A–F) and Western blotting result (Fig. 6G, H) both showed that IL-1β remarkably diminished expression of COL II and increased the levels of COL X and MMP13. Interestingly, treatment with conditioned medium from the administration of Exo-Gel, but not Gel, reversed the effects of IL-1β on COL II, COL X and MMP13.

It is now well accepted that pro-inflammatory cytokines, which activate the JAK/STAT pathway [58–62] as well as the NF-κB pathway both modulating the expression of COL II, COL X and MMP13 are critically involved in the development of OA [63–65]. The further in vitro results showed that conditioned medium from the administration of Exo-Gel decreased phosphorylation of p65 as a member of NF-κB family and STAT3 in chondrocytes treated with IL-1β (Fig. 6I, J). In summary, the above results indicate that Exo-Gel could attenuate IL-1β-triggered apoptosis and degeneration of chondrocytes through inhibiting NF-κB and STAT signaling pathway.

### Exo-Gel boosted retention of Exo in limbs

**Fig. 7:**
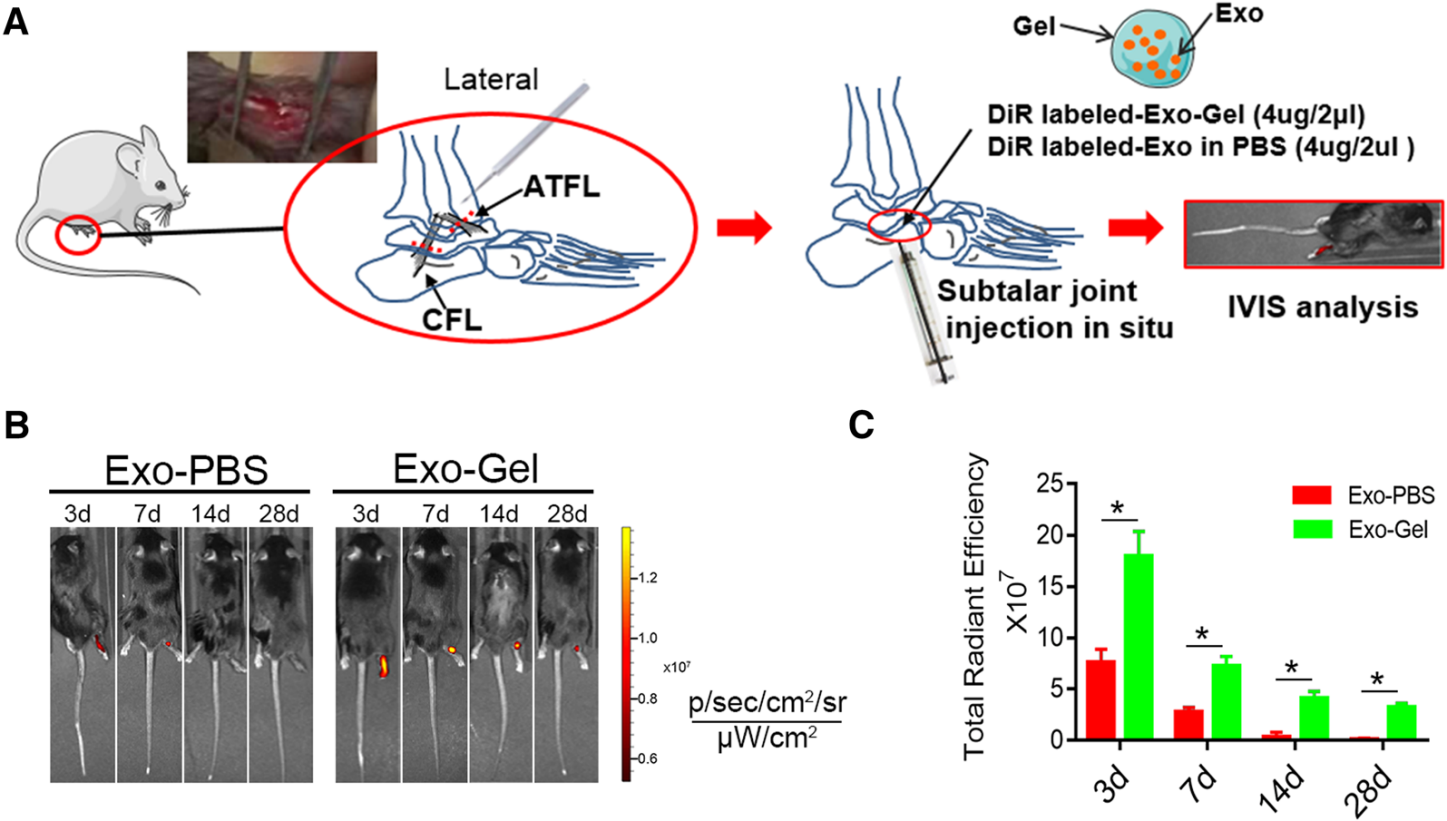
Exo-Gel promoted Exo retention in the ankle. **A** Schematic diagram of experimental design. The DiR-labeled Exo (4 µg/2 µl) was injected with or without hydrogel to subtalar joint after transecting ATFL/CFL, to establish ankle-subtalar joint complex instability. (B) Representative in vivo fluorescence imaging of ankle at 3, 7, 14, and 28 d after transplantation of Exo-Gel or Exo alone. **B** Quantitative analysis of fluorescence intensities of ankle-subtalar joint after transplantation of Gel incorporating Exo or Exo alone. Data are expressed as means ± SEM (n = 3). *p < 0.05

According to those previously described [11–13], ankle-subtalar joint complex instability was established by transecting ATFL/CFL, to induce posttraumatic STOA. The thermoresponsive Gel, due to its viscosity and hardness, provides a natural matrix barrier to lock Exo and prevents its rapid loss. In vitro, we had demonstrated that Exo-Gel served as a temporary repository for the continuous release of Exo without change biological characteristics. The DiR-labeled Exo was intra-articularly injected with or without Gel in situ to subtalar joint, and then the retention was analyzed using IVIS imaging system in vivo (Fig. 7A). The results at 3, 7, 14, and 28 d after injection revealed that compared with the joints treated with Exo alone, Exo-Gel-treated joint had a stronger fluorescent signal (representing DiR-labeled Exo) (Fig. 7B, C), indicating that Gel enhanced Exo retention in the injured joint. As we had discovered, the main disadvantage of direct injection of Exo was quick clearance from the joints, which could cause the drug concentration reduction at the site of action and limited uptake of the agents by the targeted cells [66]. Clinically, more frequent injections can result in infection, inflammation and decreased compliance. Thus, in this study, we only evaluated the therapeutic effect of Exo-Gel on chronic STOA in vivo.

### Exo-Gel alleviated STOA

**Fig. 8:**
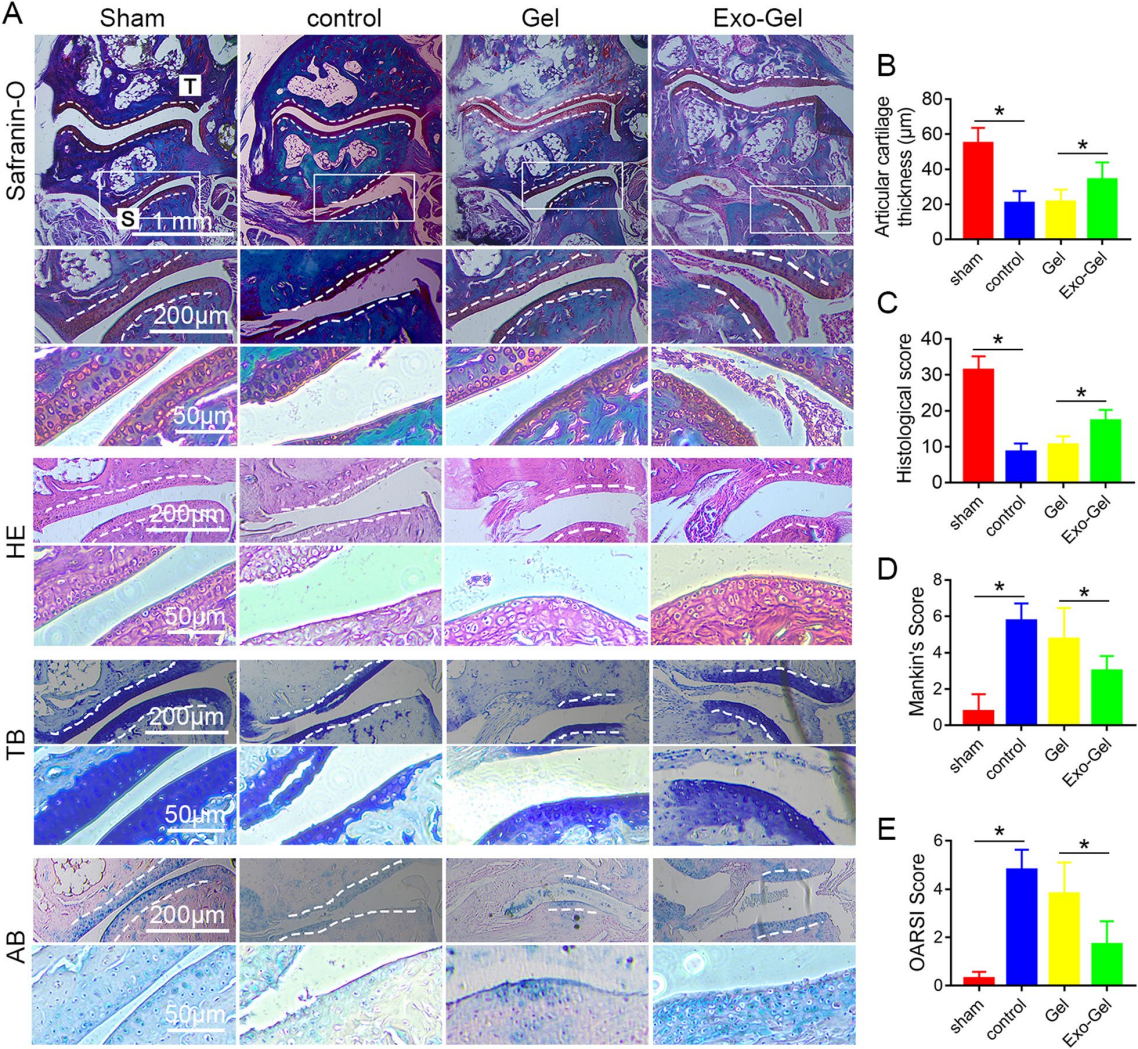
Exo-Gel alleviated STOA at 8 weeks postoperatively in mouse ankle-subtalar joint complex instability model. **A** Histological analysis was conducted of subtalar joint section with safranin-O (first row), HE (second row), TB (third row), and AB (forth row) staining to evaluate cartilage degeneration. Inset box indicated the regions shown in the enlarged images below (bar = 1 mm, 200 μm, and 50 μm). **B**. Thickness of cartilage of lateral calcaneus was measured at 15 points in every section in **A**. Data are expressed as means ± SEM (n = 4). **C** Evaluation of cartilage morphology using the Histological grading, Mankin histological, and OARSI score system. Data are expressed as means ± SEM (n = 4). *p < 0.05

At 8 weeks, safranin-O, HE, toluidine blue (TB) and AB staining were used to show the integrity of articular cartilage at mouse ankle-subtalar OA model by resection of lateral ligaments/tendons (Fig. 8A; Additional file 1: Fig. S2). Notably, in contrast to sham group, subtalar joint was degenerated at 8 weeks after the surgery while tibiotalar joint was less degenerated. In subtalar joint, lateral surface of the calcaneus was mainly affected, and severe cartilage degeneration there was observed, which is consistent with previous literature reports [13]. The cartilage in Exo-Gel group showed mild degeneration, and normal morphology and distribution of chondrocytes, with more appearance to normal cartilage than the control group or Gel group, which manifested remarkable thinning of articular cartilage, and atypical distribution of cartilage cells (Fig. 8A, B). Due to quick clearance of Exo from the joints (Fig. 7), the serious abrasion of the cartilage surface of talocrural joint could still be observed after Exo-PBS injected directly as expected (Additional file 1: Fig. S2). On the basis of the Histological grading, OARSI and Mankin histological score of subtalar joint (Fig. 8C–E) in operated and sham limbs, for quantifying the extent of cartilage injury, the data showed that Exo-Gel exhibited a more evidently protective influence on cartilage after ankle-subtalar joint complex instability, and delay occurrence of STOA.

### Exo-Gel inhibited degeneration of cartilage of subtalar joint

**Fig. 9:**
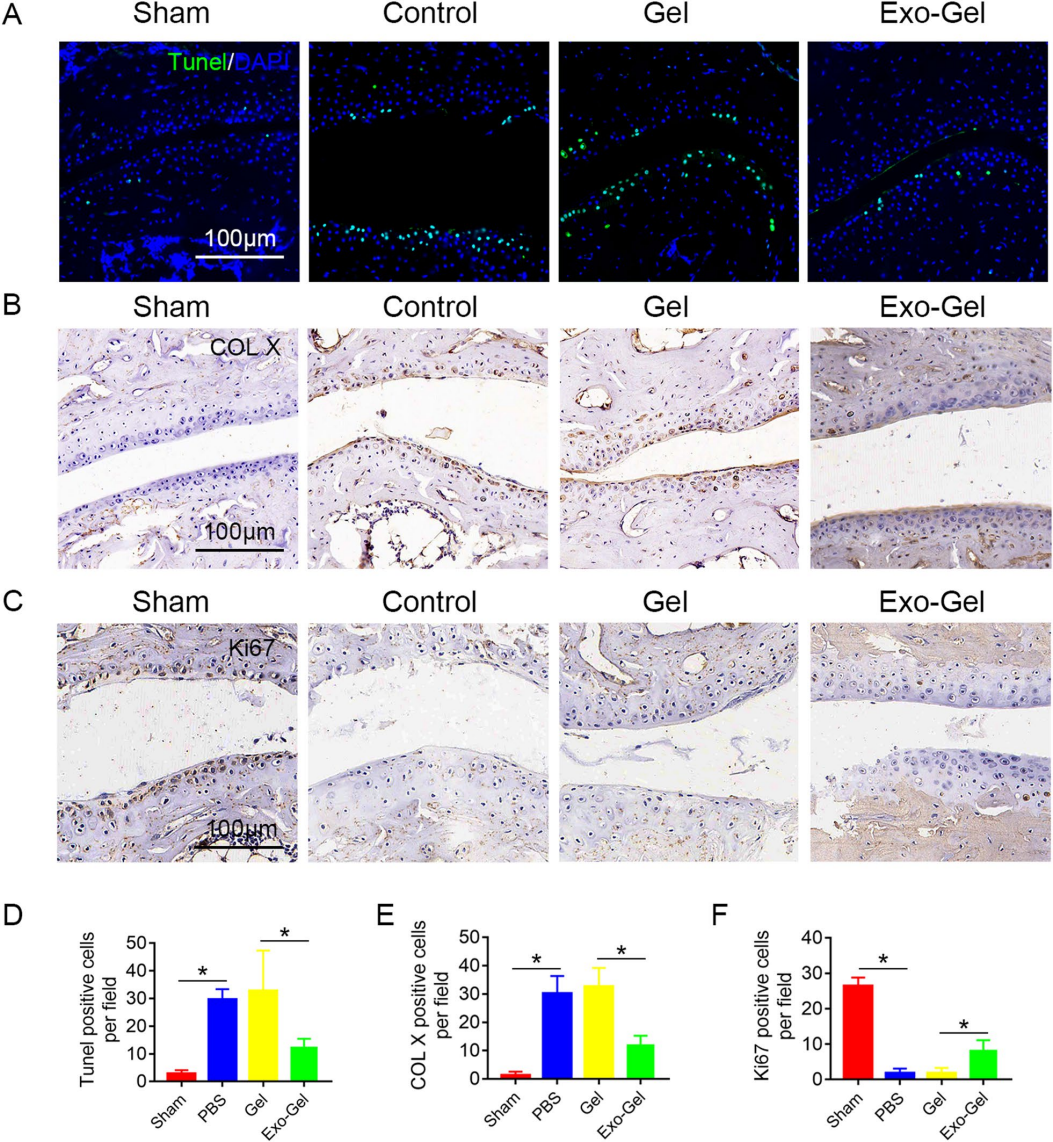
Exo-Gel inhibited cartilage degeneration of subtalar joint. **A** TUNEL (Green) staining in the subtalar joint section to analyze chondrocyte apoptosis at 4 weeks postoperatively, DAPI (blue) staining for cell nuclei (bar = 100 μm). **B**, **C** immumohistochemical staining of COL X (**B**) and Ki67 (**C**) in the subtalar joint section (bar = 100 μm). (D) Quantitative assessment of number of apoptotic cells in **A**. Data are expressed as means ± SEM (n = 4). (E, F) quantitative analysis of COL X and Ki67 positive cells in cartilage tissue in **B** and **C**, respectively. Data are expressed as means ± SEM (n = 4). *p < 0.05

Finally, we sequentially analyzed biological characteristics of chondrocytes of the ankle during the development of OA at 4 weeks postinjury [13]. TUNEL positive cells increased in control group, indicating that chondrocyte apoptosis was induced by excessive mechanical loading in the earlier stage at 4 weeks (Fig. 9A, D). In contrast to Gel, Exo-Gel reduced apoptosis of chondrocytes. Meanwhile, immumohistochemistry showed that hypertrophic chondrocytes appear in abundance, and proliferation ability of chondrocytes decreased in OA model (Fig. 9B, C, E, F). However, Exo-Gel reversed the biological characteristics of chondrocytes, including inhibiting hypertrophy and increasing proliferation in the OA model. These data indicate that attenuation of chondrocyte apoptosis and degeneration after the treatment with Exo-Gel inhibited progression of STOA.

## Discussion

Thermosensitive Gel with the good biocompatibility, as a polymer chain network, has been reported to be used as sustained release carrier of monosialoganglioside (GM1) [33]. In this study, we provided evidence that the incorporation of Exo did not affect the physical properties of thermosensitive Gel, the temperature-induced liquid-gel phase transition. Then, we found that Exo-Gel in vitro could sustain a slow-release of Exo, and did not affect the biological activity of Exo, which could provide a prolonged localized release of Exo. In vivo, our results as well highlighted that the retention of PRP-Exo in the ankle was improved by this Gel, which temporarily stored Exo and prevented fast diffusion of Exo out of the joint. These evidences provide a novel strategy of cell-free therapy in which the protection of cartilage after joint instability is reinforced from the sustained release of therapeutic PRP-Exo.

Recently, it has been suggested PRP activity might also be due to the efficient intercellular communication of bioactive molecules, mediated by nanosized vesicles, identified as Exo [20]. Besides, a high expression of marker proteins of Exo, such as CD9, CD63, and CD81 [22, 23, 67], these growth factors, including TGFβ1, PDGFBB, VEGF, and SDF-1 identified as widespread in PRP [14], could also exist encapsulated within Exo. The growth factors have a positive regulation effect on the proliferation and migration of mBMSCs and chondrocytes [24, 33, 49], and PRP-Exo have a possible physiological role as a means of delivering these growth factors across the extracellular space in tissue regeneration [68–70]. Our results verified that Exo released from Exo-Gel has the same effects with growth factors, besides that they could be taken up by cells as well as normal Exo, which prompted that Gel did not affect the biological function of Exo and its contents. Furthermore, our data indicated that inhibition of TGFβ1 by its neutralizing antibody could suppress Smad2/3, ERK1/2, and p38 signaling pathways, as considered to be the key in the regulation of chondrogenic differentiation of BMSCs [44], leading to the weakened ability of Exo-induced chondrogenic differentiation. We also found that after neutralizing TGFβ1, ERK1/2 and p38 activation level in mBMSCs treated with Exo was still higher than that in Gel group, suggesting that other growth factors are involved in the regulation of chondrogenic differentiation [45–47].

A review conducted by Andia and Maffulli [49] confirmed that growth factors present in PRP might be responsible, at least in part, for the antiinflammatory effects, partially attributable to reduced NF-κB signaling. Corroborating these findings, PDGFBB is showed to inhibit IκB kinase α (IKKα), thereby suppressing activation of NF-κB and transcription of its downstream targets involved in inflammation, cartilage degradation or chondrocyte apoptosis and reversing the antianabolic effects of IL-1β [71]. Moreover, PRP releasate completely reversed the IL-1β-induced inflammatory response of chrondrocytes isolated from patients with OA, contribute to the decrease in NF-κB activation [72], and the recovered synthesis of COL II and ACAN [72, 73]. TGF-β has been reported to prevent hypertrophy via Smad2/3 signaling, through upregulating the expression of SOX9 and downregulating the expression of runt-related transcription factor (RUNX)2 [54]. As two main chondrocyte phenotype-determining transcriptional regulators, they are reversely regulated by pro-inflammatory cytokines such as IL-1β and TNF-α [74]. Previous studies have shown that proinflammatory cytokines can also activate the STAT3 signaling pathway to induce chondrocyte damage [58–62]. Although there are few studies on the anti-inflammatory effects of PRP-Exo on chondrocytes, considering that the contents of Exo are similar to those in PRP, we believe that Exo have strong antiinflammatory effects and can inhibit inflammation-induced chondrocyte apoptosis and degeneration. Our data showed that Exo could simultaneously inhibit the activation of IL-1β-induced-NF-κB and -STAT3 in chondrocytes to play a protective effect.

The animal model of ankle sprain was first reported in 2008 [75]. Based on that study, in 2015 Chang el at. [13] elaborated the cytohistological change in the cartilage on talocrural joint and subtalar joint after different ankle ligament resection in mice. It was confirmed that the lateral ligamentectomy of ATFL and CFL only induced the occurrence of subtalar OA, and had almost no effect on talocrural joint. In 2021, Liu et al. [76] believed that the resection of the lateral ligaments would not only cause the degeneration of subtalar joint, but also affect the development of the medial talocrural OA. Although there is some controversy over whether the lateral ligament resection model can induce the degeneration of talocrural joint, it is certain that the lateral ligament resection can provide a reliable animal model of STOA. In this study, the site of ankle OA induced by lateral ligament resection model were similar to those demonstrated by Chang et al. [13] with a high probability at 8 weeks, that is, subtalar joint was affected, but not talocrural joint. However, due to the late start of the research on STOA model, compared with the knee OA, its occurrence mechanism and interventions is still rarely reported at present. Our data showed that in the process of STOA, the chondrocytes of cartilage surface, especially on the side of the calcaneus, undergone apoptosis and hypertrophy, and their proliferation ability was significantly weakened, which is similar to the changes in chondrocyte biological behavior in the process of knee OA [54–57]. However, Exo-Gel inhibited chondrocyte apoptosis and hypertrophy and promoted their proliferation, leading to alleviation of STOA. SDF-1 has been confirmed to be able to recruit endogenous MSCs to the injured area and participate in OA repair [40, 41]. Since these factors including SDF-1 and TGFβ1 are abundant in Exo, the induction of stem cell homing and chondrogenic differentiation may be another mechanism of cartilage repair.

In this study, PRP-derived Exo were incorporated in thermosensitive Gel as a sustained delivery system. Our study demonstrated that PRP-Exo had positive regulation of mBMSCs function and played an protective role against IL-1β-induced apoptosis and degeneration of chondrocytes in vivo. Application of this system allowed retention of Exo in the ankle compared to Exo only injection, and consequently maintained the local concentration of Exo. Together, Exo-Gel have effective effects on suppressing cartilage degeneration and development of STOA.

## Supplementary Information

** Additional file 1: Figure S1.** Identify of mBMSCs. **Figure S2.** Exo-PBS did not prevent the progress of STOA.

## Data Availability

The datasets used and/or analyzed during the current study are available from the corresponding author on reasonable request.

## Abbreviations

Exo: Exosomes
PRP: Platelet-rich plasma
PRP-Exo: Platelet-rich plasma-derived exosomes
Hydrogel: Gel
Exo-Gel: Platelet-rich plasma-derived exosomes incorporated Hydrogel
OA: Osteoarthritis
STOA: Subtalar osteoarthritis
ATFL: Anterior talofibular ligament
CFL: Calcaneal fibular ligament
mBMSCs: Mouse bone mesenchymal stem cells
TGFβ1: Transforming growth factor β1
PDGFBB: Platelet derived growth factor BB
VEGF: Vascular endothelial growth factor
SDF-1: Stromal cell derived factor-1
COL II: type II Collagen
COL X: type X Collagen
ACAN: Aggrecan
NF-κB: Transcription factor nuclear factor kappa B
MMP13: Matrix metallopeptidase 13
APP: Activated platelet pellet
TEM: transmission electron microscopy
SEM: Scanning electron microscopy
NTA: Nanosight tracking analysis
AB: Alcian blue
TB: Toluidine blue (TB)
