# Supplementary material for: Exosomes derived from platelet-rich plasma administration in site mediate cartilage protection in subtalar osteoarthritis
Source: J Nanobiotechnology. 2022 Jan 29;20:56. doi: 10.1186/s12951-022-01245-8 (PMC8801111; doi:10.1186/s12951-022-01245-8)
Supplement: Supplementary file 1 — Additional file 1: Figure S1. Identify of mBMSCs. Figure S2. Exo-PBS did not prevent the progress of STOA. [file 12951_2022_1245_MOESM1_ESM.docx]

**
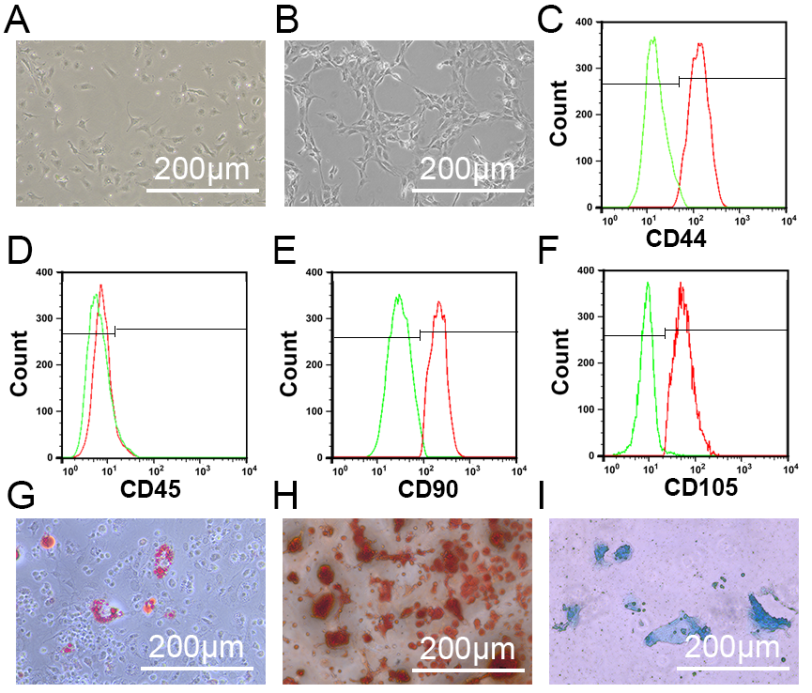
**

**Supplementary Figure 1. Identify of mBMSCs.** (A, B) mBMSCs exhibited a typical spindle-shaped morphology. The morphology of BMSCs was round, spindle, polygonal at P0 and became long fusiform at P3. Scale bar: 200μm. (C-F) Flow cytometric analysis of the surface markers in mBMSCs (95.32% CD44, 2.34% CD45, 97.21% CD90 and 96.23% CD105). (G-I) mBMSCs displayed ability of adipogenic differentiation (Oil red O staining), osteogenic differentiation (Alizarin red staining), and chondrogenic differentiation (AB staining). scale bar: 200 μm.


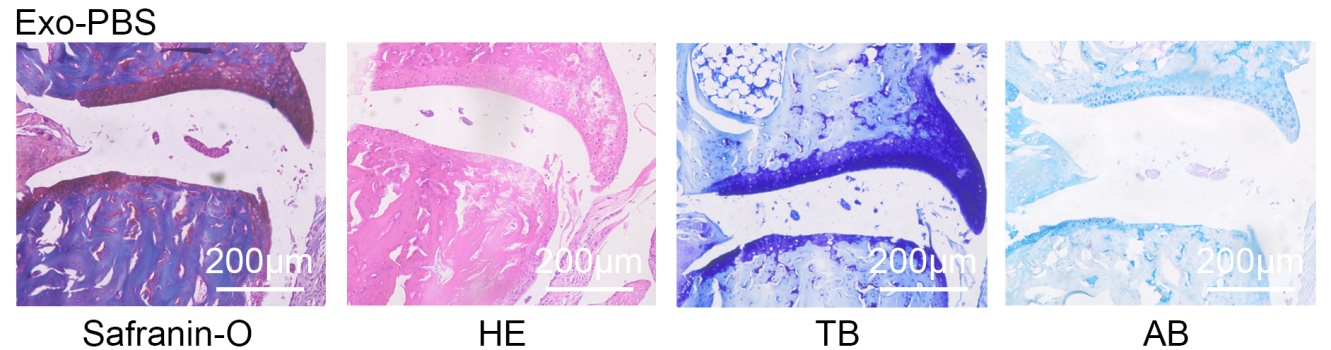
**Supplementary Figure 2. Exo-PBS did not prevent the progress of STOA.** Subtalar joint section with safranin-O, HE, TB, and AB staining showed the serious abrasion of the cartilage surface of talocrural joint, especially on the side of the calcaneus (bar = 200μm).
